# What Do Nurses Value Most? Evidence From a Discrete Choice Experiment in Indonesia’s Remote Regions

**DOI:** 10.34172/ijhpm.9538

**Published:** 2026-06-23

**Authors:** Ferry Efendi, Eka Mishbahatul Mar’ah Has, Rifky Octavia Pradipta, Mei Chan Chong, Anna Kurniati

**Affiliations:** ^1^Faculty of Nursing, Universitas Airlangga, Surabaya, Indonesia.; ^2^School of Nursing and Midwifery, La Trobe University, Melbourne, VIC, Australia.; ^3^Research Center in Advancing Community Healthcare (REACH), Surabaya, Indonesia.; ^4^Kaohsiung Medical University, Kaohsiung, Taiwan.; ^5^Department of Nursing Science, Faculty of Medicine, University Malaya, Kuala Lumpur, Malaysia.; ^6^Ministry of Health, Jakarta, Indonesia.

**Keywords:** Nurse Distribution, Access to Healthcare, Discrete Choice Experiment, Nurses, Health Workforce, Job Creation

## Abstract

**Background:**

The uneven distribution of nurses across Indonesia’s remote regions remains a major obstacle to achieving universal health coverage and Sustainable Development Goals. Recruitment and retention policies for remote areas are often based on assumptions about what nurses value, while empirical evidence remains limited. This study aimed to examine the job attributes influencing nurses’ preferences for working in remote and very remote areas in Indonesia.

**Methods:**

A discrete choice experiment (DCE) was conducted among 243 nurses with at least one year of experience in remote healthcare facilities in East Nusa Tenggara, Papua, and West Papua. Job attributes and levels were identified through literature reviews and focus group discussions (FGDs). Six attributes were included: work location, employment status, supporting facilities, basic living facilities, student financial aid, and monthly net income. Preferences were estimated using a conditional logit model, with willingness-to-accept (WTA) estimates supporting policy interpretation.

**Results:**

Basic living facilities were the most influential factor in shaping nurses’ job preferences. Positions offering reliable access to clean water, electricity, and internet services were significantly more likely to be chosen than those without such facilities (odds ratio [OR] = 2.06; 95% CI: 1.82–2.33). Jobs in very remote areas were also preferred over rural postings (OR = 1.76; 95% CI: 1.52–2.04). In contrast, civil servant employment was less preferred than contract-based positions (OR = 0.56; 95% CI: 0.49–0.64), and direct provision of housing and transportation did not significantly influence job choice (OR = 1.07; 95% CI: 0.94–1.22). A higher monthly income increased the likelihood of selecting a remote posting.

**Conclusion:**

Nurses’ preferences for remote postings in Indonesia are driven mainly by living conditions, employment security, and location, rather than financial incentives alone. Policies should prioritize basic infrastructure improvements and stable employment arrangements.

## Introduction

**Key Messages**

**Implications for policy makers**

- Improving reliable access to basic living facilities, particularly clean water, ‎electricity, and Internet services, may increase the attractiveness of remote ‎postings for nurses.‎
- Contract-based employment arrangements may warrant further consideration in ‎workforce policies, given respondents’ stronger stated preferences for these ‎positions compared to other employment arrangements.‎
- Recruitment packages for remote areas should prioritize practical and context-‎appropriate support, as financial allowances alone may be less attractive than ‎direct support in remote settings.‎
- While improving rural infrastructure and employment arrangements may enhance ‎the attractiveness of remote postings, the implementation of such measures must ‎consider existing resource, infrastructure, and logistical constraints in remote ‎regions of Indonesia.‎

**Implications for the public**

Ensuring adequate health services in remote and rural communities depends partly ‎on the ability to attract and support health workers. This study highlights the job ‎attributes that respondents consider important when evaluating remote postings, ‎particularly access to basic living conditions and employment stability. When ‎nurses have reliable housing, utilities, and clearer employment arrangements, ‎remote postings may become more acceptable and sustainable options. However, ‎improving these conditions is challenging. Structural constraints, limited ‎infrastructure, and resource shortages remain significant barriers in many remote ‎areas of Indonesia. Addressing these challenges requires coordinated efforts ‎across health, infrastructure, and administrative sectors to support nurses and ‎improve access to essential healthcare services for communities living in ‎underserved areas.‎

Achieving Sustainable Development Goals and advancing universal health coverage require an adequate and well-distributed health workforce. The World Health Organization (WHO) estimates that approximately 4.45 skilled health professionals, including physicians, nurses, and midwives, are needed per 1000 population to deliver essential health services.^1^ However, the maldistribution of health workforce remains a global challenge. Globally, only approximately 38% of nurses work in rural or remote areas.^2^ In Indonesia, disparities in nurse distribution are particularly pronounced. In 2021, fewer than 4% of nurses worked in remote areas (18 650 of 511 191 nationwide).^3^ The nursing workforce is heavily concentrated in Java and Sumatra, while provinces such as Papua, East Nusa Tenggara, and Maluku classified as remote continue to face substantial shortages.^3^ Indonesia has set a national target of two nurses per 1000 population by 2025; however, the current nurse density remains among the lowest in the Asian region.^4,5^ Only a small number of provinces, primarily urban areas in Java and Bali, currently meet the WHO-recommended threshold of 2.28 nurses per 1000 population.^6^

 Nurses play a central role in delivering essential healthcare services in rural and remote regions; however, recruiting and deploying nurses to these settings remains challenging. Persistent shortages are shaped by multiple factors, including inequitable resource distribution, limited infrastructure, and weaker employment incentives in remote areas.^7,8^ Low compensation, limited benefits, professional isolation, restricted access to education and health services, and unfamiliar cultural environments may further discourage nurses from accepting or remaining in remote postings.^9-11^ Evidence from prior research has also identified leadership, work environment, career development opportunities, salary, and organizational support as key determinants of nurses’ job satisfaction and workforce stability. These factors may be particularly salient in remote contexts where employment conditions are often more constrained.^12^ Structural challenges, such as insufficient human resource planning and low training enrolment, also contribute to workforce shortages, which are further compounded by population growth and high turnover rates.^13,14^ The COVID-19 pandemic has intensified these challenges, disproportionately affecting rural and remote regions.^15^

 In response, the Indonesian government has implemented several initiatives aimed at improving the recruitment and deployment of health workers in underserved communities, including national placement programs.^2,16,17^ Despite these efforts, sustaining an adequate nursing workforce in remote settings remains difficult.^11^ Understanding nurses’ needs and expectations is therefore an important component of workforce policy development.^18^ However, empirical evidence on how nurses value different job attributes in remote contexts remains limited in Indonesia.

 The discrete choice experiment (DCE) studies offer a structured approach to examining stated preferences for job characteristics by requiring respondents to make trade-offs between alternative employment attributes.^19^ DCEs have been widely applied to health workforce research to inform recruitment and deployment strategies.^20,21^ Previous studies have examined preferences among medical, nursing, and midwifery students in Indonesia,^22^ as well as among nurses and midwives in other low- and middle-income settings, including Peru^21^ and Timor-Leste.^23^ These studies highlight the relevance of employment conditions, incentives, and living environments in shaping workforce decisions. Building on this literature, the present study applies a DCE framework to examine nurse stated preferences for job attributes associated with recruitment in remote areas of Indonesia. However, despite existing DCE studies in other settings and among health students in Indonesia, empirical evidence remains limited regarding the relative importance of job attributes influencing practicing nurses’ preferences for working in remote and very remote areas in Indonesia. This study aimed to examine job attributes influencing nurses’ preferences for working in remote and very remote areas in Indonesia.

## Methods

###  Study Design

 This study used a cross-sectional design incorporating a DCE to examine the job attributes influencing nurses’ recruitment preferences for remote postings. In the DCE, respondents were asked to choose between hypothetical job scenarios composed of different combinations of job attributes and levels, reflecting alternative employment options in remote settings.^24^

###  Participant and Sampling

 The study was conducted in public health center facilities and hospitals located in remote regions across three Indonesian provinces: East Nusa Tenggara, Papua, and West Papua. The study sites were selected based on the official classification of remote areas defined by the Decree of the Directorate General of Health Services (No. HK.02.02/11/0373/2019)^25^ as well as the distribution of nursing personnel within these regions. The decree identified all public health centers and hospitals located in remote areas across these three provinces in Indonesia.

 Data collection took place in August 2023. A non-probability purposive sampling approach was applied, participants were registered nurses who had worked for at least one year in a remote area in current posting, either in public health center (*Puskesmas*) or hospitals. Recruitment was conducted through the respective operational units of each health facility; nurses interested in participating were invited to contact the enumerators, who were present onsite at the placement locations for one week. Sample size for the DCE was guided by WHO (2012) recommendations indicating that a minimum of 30 respondents is acceptable for estimating main effects in DCE studies. We recruited 243 nurses, far exceeding this threshold and providing sufficient precision to estimate main effects using the conditional logit model.^19^ Nurses were approached in person by trained enumerators and invited to participate voluntarily, resulting in a final sample of 243 nurses.

###  Attribute and Levels Development

 Job attributes and levels were identified through a targeted literature review followed by focus group discussions (FGDs). The literature search was conducted in Scopus, Web of Science, EBSCO, ProQuest, and PubMed using combinations of the keywords “recruitment,” “attract,” “rural,” “remote,” “nurse,” and “discrete choice experiment” or “DCE.” The search was limited to peer-reviewed articles published in English between 2012 and 2022. A preliminary list of attributes was compiled and screened based on policy relevance, contextual applicability to Indonesia, and feasibility for inclusion in a DCE. Two FGDs were undertaken as part of the formative qualitative phase to develop attributes and levels for the DCE. The first FGD brought together senior nurses from health facilities in underserved remote areas alongside academics with expertise in service delivery in disadvantaged settings. This session generated an initial list of 15 candidate attributes that were considered potentially relevant for inclusion in the DCE survey (Table 1).

**Table 1:** Attributes and Levels of Discrete Choice Experiment

**Attribute**	**Levels**
Location	Rural*
	Remote
	Very remote
Employment status	Civil servant
	Permanent non-civil servant
	Contract*
Supporting facilities	Housing and transportation (physical)
	Housing and transportation as an allowance (financial)
	None*
Basic living facilities	No issues with clean water, electricity, and internet
	Periodic/difficulties with clean water, electricity, and internet
	None*
Student financial aid	Government scholarship
	Self-funded*
Net income (in a month)	IDR 10 million^a^
	IDR 15 million^a^
	IDR 20 million^a^

Description: *level of comparison or control. ^a^1 USD ≈ IDR 15 694 (exchange rate, November 2023).

 A second FGD was subsequently conducted with the same participants to refine, consolidate, and prioritise the candidate list, with the explicit aim of reaching consensus on the final set of attributes and their operational definitions.

 Six attributes were retained: work location, employment status, supporting facilities, basic living facilities, student financial aid, and monthly net income. Employment status comprises three categories: civil servant, referring to government-employed nurses with civil servant status; permanent non-civil servant, referring to permanently employed nurses without civil servant status; and contract, referring to nurses employed under a fixed-term or temporary work agreement. Five attributes were non-monetary and one was monetary. Attribute levels were designed to reflect realistic employment conditions under existing placement schemes and compensation structures. Income levels were selected to represent plausible monthly earnings for nurses in remote areas and were pilot tested to ensure credibility and comprehension. The survey targeted registered nurses employed in public and private health facilities, including hospitals and public health centers. Respondents varied in age, years of experience, employment status, and prior exposure to rural or remote settings, allowing the study to capture preferences across diverse professional backgrounds. Non-monetary attributes were operationalized as categorical variables, while monthly net income was included to enable the estimation of trade-offs between financial and non-financial job characteristics.

###  Discrete Choice Experiment Questionnaire Design

 After finalizing the attributes and levels, a structured DCE questionnaire was developed. Of the six attributes, five consisted of three levels, and one consisted of two levels. An orthogonal fractional factorial design was used to generate an efficient set of choice scenarios for the experiment. The experimental design was implemented using R statistical software and produced 18 choice sets, each containing two hypothetical job alternatives. An opt-out option was intentionally excluded to encourage respondents to make explicit trade-offs between job attributes. The questionnaire consisted of three sections: (1) respondents’ sociodemographic and professional characteristics; (2) an instructional section that included a “cheap talk” script and clear explanations of the attributes and levels; and (3) the DCE task, comprising 18 choice scenarios presented sequentially, with each scenario displayed on a separate page. Prior to the main survey, the questionnaire was pilot-tested with 45 nurses to assess clarity, comprehension, and feasibility. Feedback from the pilot study indicated that the instrument was understandable and appropriate for the target population (Figure).

**Figure:**
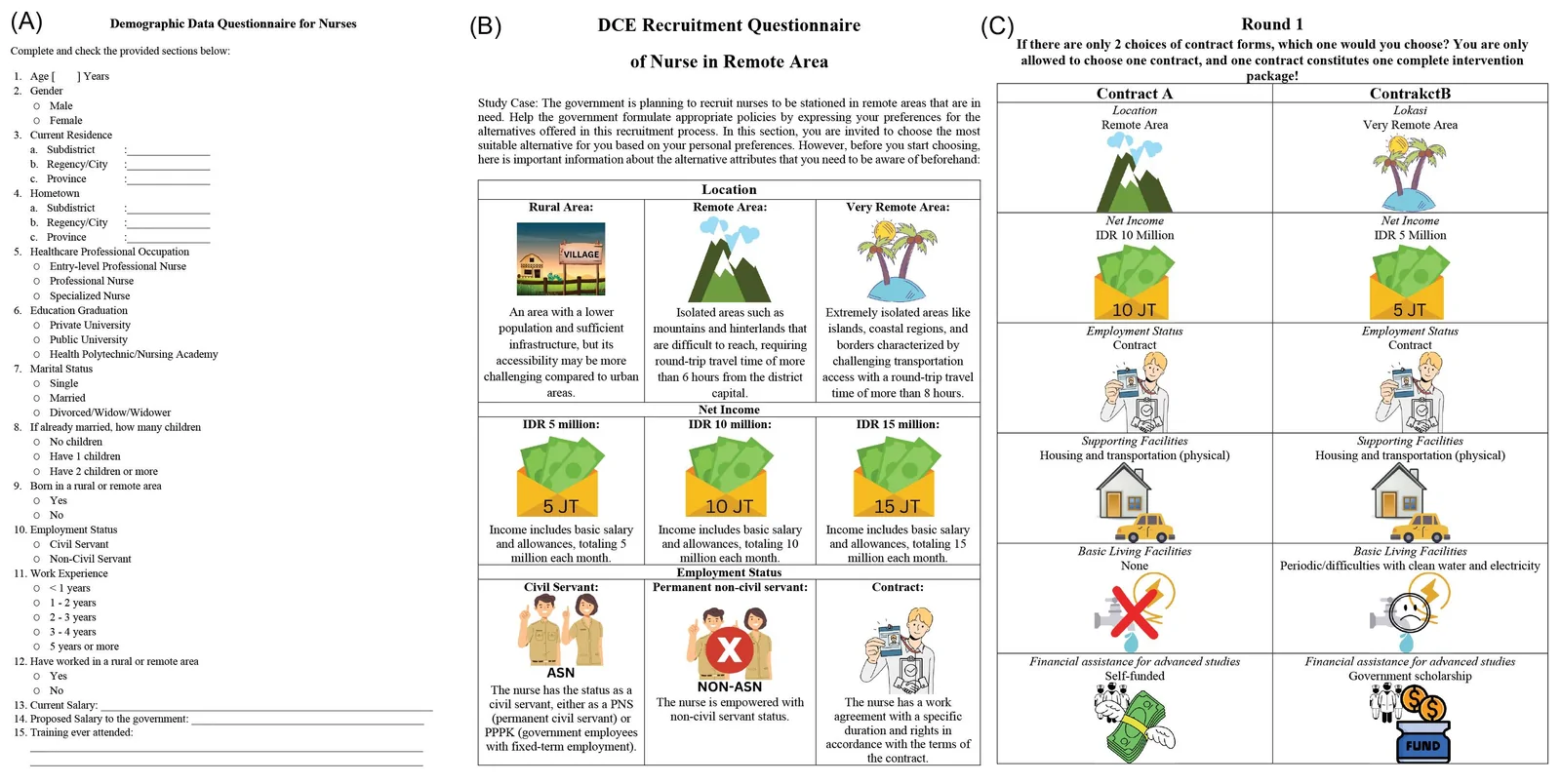
The Nurse Recruitment Questionnaire. (A) Respondent characteristics; (B) cheap talk script; and (C) example of a choice task from the 18-choice-set DCE recruitment section. Abbreviations: DCE, discrete choice experiment; ASN, civil servant.

###  Data Analysis 

 Conditional logit was selected because the study aims to estimate the average effect of job attributes on nurses’ choices under random utility maximization, and the attributes vary across alternatives within each choice set consistent with the conditional logit framework for DCE data.^18^ In addition, it provides a parsimonious and statistically stable baseline model with straightforward interpretation of coefficients/odds ratios (ORs) and transparent derivation of marginal willingness-to-accept (mWTA) from the estimated parameters.

 Willingness-to-accept (WTA) estimates were calculated by dividing the coefficients of non-monetary job attributes by the coefficient of the income attribute. These estimates represent the additional monetary compensation required for respondents to accept less preferred job conditions and were used to support policy-relevant interpretation of trade-offs between financial and non-financial attributes.^28^ Although the calculation follows the same ratio-based approach commonly used to estimate willingness-to-pay in DCE studies, the term WTA was adopted to reflect the compensatory framing of income in an employment context rather than an out-of-pocket payment. mWTA was computed as the negative ratio of each attribute coefficient to the coefficient of the monetary (compensation) attribute. The resulting estimate was interpreted as the additional compensation required to accept a less preferred job attribute and was expressed in the same monetary unit as the compensation variable (with currency conversion applied, where relevant).^28^ To improve readability for an international audience, income levels were converted from Indonesian Rupiah (IDR) to United States dollar (USD) using the average exchange rate in 2023 (1 USD ≈ IDR 15 694; exchange rate, November 2023).

## Results

###  Respondent Profiles

Table 2 summarizes the sociodemographic characteristics of the 243 participating nurses. The mean age of the respondents working in remote healthcare facilities was 33.2 years (standard deviation [SD] = 5.4). Most participants were female (58.44%), and 59.26% held professional nursing qualifications. Over half of the respondents (51.85%) graduated from health polytechnics or nursing academies. The majority were married (67.90%), and nearly half reported having more than two children (46.91%). A substantial proportion of respondents originated from rural or remote areas (67.90%). In terms of employment status, slightly more than half were civil servants (51.03%). Most respondents reported more than four years of work experience (69.55%), while the remainder had between one and four years. The majority had prior experience working in rural or remote areas (82.72%). Respondents were recruited from healthcare facilities in East Nusa Tenggara, Papua, and West Papua. However, province-level identifiers were not retained in the final analytic dataset, which precluded descriptive reporting and comparison of respondent characteristics by province. Therefore, the analysis focused on aggregate preference patterns among nurses working in remote settings.

**Table 2:** Respondent Characteristics (n = 243)

**Variable**		**n**	**%**
Age (y)	Mean (SD)	33.2 (5.4)	
Sex	Male	101	41.56
	Female	142	58.44
Healthcare professional occupation	Professional nurse	144	59.26
	Entry-level professional nurse	99	40.74
Education graduation	Public university	27	11.11
	Private university	90	37.04
	Health polytechnic/nursing academy	126	51.85
Marital status	Single	77	31.69
	Married	165	67.90
	Divorce/widower	1	0.41
Number of children	No children yet	92	37.86
	1 Child	37	15.23
	‎2 or more children‎	114	46.91
Born in a rural or remote area	Yes	165	67.90
	No	78	32.10
Employment status	Civil servant	124	51.03
	Non-civil servant	119	48.97
Work experience	1-2 years	32	13.17
	>2-3 years	38	15.64
	>3-4 years	24	9.88
	>4 years	149	61.32
Have worked in a rural or remote area in previous assignment	Yes	201	82.72
	No	42	17.28
	Total	243	100

Abbreviation: SD, standard deviation.

###  Preferences for Recruitment of Nurses in Remote Areas

 The Conditional logit estimates were interpreted relative to the predefined reference categories shown in Table 3. The conditional logit model demonstrated an acceptable fit (log likelihood = −1559.623; Rho^2^ = 0.501; Akaike information criterion = 3147.245). Overall, nurses’ job preferences were shaped more strongly by non-financial job attributes than financial incentives alone. For work location, compared with rural postings, respondents showed stronger preferences for positions in remote (OR = 1.50; 95% CI: 1.32–1.70) and very remote areas (OR = 1.76; 95% CI: 1.52–2.04), suggesting that remoteness itself was not necessarily perceived as a deterrent when other job conditions were favorable. The corresponding negative mWTA estimates suggest that respondents were willing to accept lower income in exchange for postings in remote and very remote areas relative to rural areas.

**Table 3:** Conditional Logit Estimation Result for Nurses Recruitment in Remote Areas

**Attribute**	**Level**	**OR (95% CI)**	**mWTA **
Location (rural area)	Remote area	1.4990 (1.32–1.70)	−US $162.7
	Very remote area	1.7570 (1.52–2.04)	−US $226.4
Employment status (contract)	Civil servant	0.5580 (0.49–0.64)	US $234.7
	Permanent non-civil servant	0.7631 (0.67–0.87)	US $108.7
Supporting facilities (none)	Housing and transportation (physical)	1.0740 (0.94–1.22)	−US $28.6
	Housing and transportation as an allowance (financial)	0.7126 (0.62–0.82)	US $136.3
Basic living facilities (none)	No issues with clean water, electricity, and internet	2.0600 (1.82–2.33)	−US $290.6
	Periodic/difficulties with clean water, electricity, and internet	0.9495 (0.83–1.08)	US $20.8
Student financial aid (self-funded)	Government scholarship	1.0600 (0.95–1.18)	−US $23.5
Net income	Net income	1.0000	
Model fit statistics	Log likelihood (null model) = −3124.453		
	Log likelihood (final model) = −1559.623		
	Rho^2^ = 0.501		
	Adjusted Rho^2^ = 0.496		
	AIC = 3147.245		
	BIC = 3230.587		
	Number of estimated coefficients = 10		

Abbreviations: OR, odds ratio; CI, confidence interval; mWTA, marginal willingness-to-accept; BIC, Bayesian information criterion; AIC, Akaike information criterion. Note: USD conversion is based on the average 2023 exchange rate (1 USD ≈ IDR 15 694) as of November 12, 2023.

 Employment status was also associated with job preferences. Contract-based positions were used as the reference category in the analyses. Compared with contract-based positions, civil servants (OR = 0.56; 95% CI: 0.49–0.64) and permanent non-civil servant arrangements (OR = 0.76; 95% CI: 0.67–0.87) were less likely to be selected. The positive mWTA values indicate the additional income required to ‎compensate respondents for accepting these employment ‎arrangements compared with contract positions‎.

 Preferences for support facilities depended on the type of support provided. Financial allowances for housing and transportation were less preferred than having no additional support (OR = 0.71; 95% CI: 0.62–0.82), whereas direct provision of housing and transportation was not significantly associated with job choice (OR = 1.07; 95% CI: 0.94–1.22). The positive mWTA indicates that respondents would require approximately US $136 in additional monthly income to accept allowance-based support.

 Regarding basic living facilities, positions offering stable access to clean water, electricity, and internet services were substantially more likely to be selected than postings without such facilities (OR = 2.06; 95% CI: 1.82–2.33). The corresponding negative mWTA estimate suggests that respondents were willing to trade some of their monthly income for reliable infrastructure and living conditions. In contrast, posts with periodic disruptions in basic services were not clearly associated with job choice relative to the reference category (OR = 0.95; 95% CI: 0.83–1.08), with only a small corresponding mWTA.

 Self-funded arrangements were used as the reference category for student financial aid. Government scholarships were not significantly associated with job choice (OR = 1.06; 95% CI: 0.95–1.18), with only a small corresponding mWTA (−US $23.5). Across attributes, negative mWTA values indicate that respondents were willing to forego income to obtain more preferred job characteristics, whereas positive values represent the additional compensation required to offset less preferred conditions. Therefore, these estimates quantify the relative trade-offs between monetary and non-monetary job attributes. Among all attributes, reliable basic living facilities emerged as the strongest driver of job choice, with the largest effect size and mWTA estimate, indicating a substantial willingness to trade income for stable access to essential infrastructure facilities. In contrast, financial allowances alone did not increase job attractiveness, suggesting that structural and living conditions may outweigh purely monetary incentives in shaping recruitment preferences. Taken together, these findings underscore the central role of non-financial job attributes in influencing nurses’ decisions to accept postings in remote and very remote areas.

## Discussion

 This study examined nurses stated preferences for job attributes associated with remote postings in Indonesia using a DCE approach. Overall, the findings indicate that several non-financial job attributes were associated with job choice, with reliable basic living facilities and very remote postings showing the largest estimated associations, alongside financial incentives.

 Nurses place a high priority on access to basic necessities, clean water, electricity, and the Internet, particularly in remote locations where healthcare infrastructure is often limited.^29^ Beyond workforce availability, reliable utilities and essential inputs are important enablers of effective service delivery.^30^ Evidence from India has similarly emphasized the role of infrastructure in progress toward health-related development goals.^31^ Limited resources and fewer opportunities for professional development in rural areas may also constrain nurses’ ability to maintain competencies and keep pace with developments in nursing practice and science.^32^ In this context, digital platforms can support information exchange and continuing learning, and a Canadian study reported that high-speed internet was an important channel for maintaining up-to-date knowledge.^33^ Technology-enabled care models may also help extend specialist support to underserved settings and respond to the particular needs of rural populations.^34^ Taken together, these considerations suggest that investments in basic living facilities can be viewed not only as material support for nurses, but also as part of the enabling environment for health service delivery in remote areas.^29,30^

 The influence of the work location should be interpreted carefully. The DCE results indicate a marginal preference for very remote postings relative to the rural reference category under controlled experimental conditions, rather than an absolute preference for more isolated areas. Nevertheless, this pattern may reflect that, when other job conditions are acceptable, geographic remoteness is not necessarily a deterrent. Providing care in remote environments often requires nurses to work alone or in small teams, which can create both opportunities and challenges for the nurses.^35^ In such settings, collaboration with other health workers can be important for service delivery and interprofessional learning.^36^ Individual backgrounds and social ties such as rural upbringings, familial connections, professional fulfillment, and a sense of belonging may also shape WTA rural or remote postings.^37^ In Indonesia, the Nusantara Sehat initiative places nurses in selected rural areas and offers incentives that may differ by remoteness.^38^ These contextual features may help explain why marginal preferences by location can vary, and why location effects should be interpreted alongside broader living and employment conditions.^37,38^

 Employment status also influenced the preferences. Contract employment was preferred relative to permanent non-civil servant arrangements, which may relate to how contract schemes are structured within programs such as Nusantara Sehat, including the perceived clarity of terms, benefits, and duration.^38^ A similar preference for government-provided contract positions has been reported elsewhere, including a study in Tehran describing “Rasmi contract” arrangements.^39^ Contract positions may be perceived as offering flexibility and openness to future opportunities, allowing professionals to gain experience while maintaining mobility across roles and settings.^40^ These patterns highlight that “job security” may be experienced differently in practice, and that employment arrangements may be valued not only for permanence but also for predictability and career control.^39,40^

 Preferences related to supporting facilities further illustrate the importance of how incentives are delivered in remote contexts. Reluctance to accept housing and transportation support in cash form may reflect the scarcity or absence of services and markets in remote areas, where money cannot easily be converted into practical solutions. In contrast, direct provision may be perceived as more reliable and effective. Similar patterns have been reported among health workers in rural Zambia, where official housing was associated with substantially stronger recruitment effects than cash-based allowance.^41^ A comparable preference for housing supplied rather than housing allowances was also noted in a survey of nursing students in Uganda.^42^ In Indonesia, regulations mandate accommodation support for health personnel in public health center facilities, although implementation may vary across areas based on local resources and conditions.^43,44^ These findings suggest that policy packages should pay close attention to feasibility and delivery mechanisms, rather than assuming that financial allowances will function as effective substitutes in remote settings.^41,44^

 On the monetary side, monthly net income was positively associated with job choice, consistent with prior evidence that pay matters for early career and practicing nurses.^45^ However, income effects should be interpreted in light of model specification and scaling; an OR close to unity for a continuous income variable can reflect units of measurement rather than the absence of an effect. Higher pay may also be viewed as compensation for higher living costs, travel burdens, and greater work demands in remote settings.^46^ At the same time, this study’s results underscore that financial incentives alone may not be sufficient if non-monetary conditions are inadequate. Evidence from Peru similarly suggests that “bundled” packages combining pay and non-financial supports, such as training opportunities, can be more persuasive than single-component incentives.^47^ Taken together, these findings point to the importance of designing balanced packages that address both financial and practical barriers to remote postings.^45,47^

 Several methodological constraints should be acknowledged when interpreting these findings. This study used a conditional logit model that estimates average preferences and does not examine preference heterogeneity across individuals or contexts. In addition, the absence of an opt-out option may increase hypothetical bias by requiring respondents to choose between job alternatives, even if neither would be acceptable in practice. As a cross-sectional study relying on self-reported choices, the findings reflect preferences at a single point in time and may not capture how preferences evolve with changing circumstances. Mixed-method approaches and longitudinal designs may help strengthen future work, including exploring how nurses’ preferences shift with experience, policy changes, and local conditions. Finally, as with all DCEs, the results represent stated preferences under hypothetical scenarios and should not be interpreted as direct evidence of retention or performance. A limitation of this study is that we estimated average preferences using a conditional logit model and did not examine preference heterogeneity through subgroups (eg, gender, rural/remote background).

## Conclusions

 This study shows that nurses stated preferences for remote postings in Indonesia are shaped more by non-monetary attributes, particularly access to basic living amenities, than by salary alone, although income remains relevant. Given the financial, administrative, and infrastructural constraints in remote settings, prioritizing improvements in essential living conditions alongside clear, time-bound contractual arrangements is likely to yield greater gains in recruitment than salary increases. These findings should be interpreted cautiously, as the study is cross-sectional, captures average stated preferences without accounting for heterogeneity, and reflects hypothetical rather than observed behavior. Nevertheless, they provide a concise and policy-relevant basis for designing more feasible recruitment strategies for Indonesia’s remote health workforce.

## Disclosure of artificial intelligence (AI) use

 The AI was utilized for grammar refinement‎.

## Ethical issues

 This study was approved by the Ethics Committee of the Faculty of Nursing at ‎Universitas Airlangga (Number: 2847-KEPK). Before taking part in the study, participants ‎were thoroughly informed about it and provided informed consent. Respondents ‎participated voluntarily and gave their verbal and written consent.

## Conflicts of interest

 Authors declare that they have no conflicts of interest.
